# Personalized risk score for post‐COVID‐19 condition: Bayesian directed acyclic graphic approach

**DOI:** 10.1111/risa.70072

**Published:** 2025-07-06

**Authors:** Sam Li‐Sheng Chen, Chen‐Yang Hsu, Tin‐Yu Lin, Amy Ming‐Fang Yen, Tony Hsiu‐Hsi Chen

**Affiliations:** ^1^ School of Oral Hygiene, College of Oral Medicine Taipei Medical University Taipei Taiwan; ^2^ Department of Emergency Dachung Hospital Miaoli Taiwan; ^3^ Master of Public Health Program, College of Public Health National Taiwan University Taipei Taiwan; ^4^ Institute of Health Data Analytics and Statistics, College of Public Health National Taiwan University Taipei Taiwan

**Keywords:** Bayesian, post‐COVID‐19 condition, risk assessment

## Abstract

Post‐COVID‐19 condition (PCC) has gained traction currently in the post‐pandemic era. To address this, we utilized a Bayesian directed acyclic graphic (DAG) model to develop a personalized composite risk score (CRS) for PCC, based on the tabular data derived from a comprehensive meta‐analysis. Our risk assessment model incorporates 215 combinations of risk factors, including personal demographic and health‐related profiles, across 41 studies involving over 860,000 COVID‐19 cases. The CRS ranges from 0 to 500, categorizing patients into risk quartiles and estimating PCC probability across SARS‐CoV‐2 variants of concerns, including Wild/D614G/Alpha, Delta, and Omicron BA.1/BA.2. External validation demonstrated accurate predictions, though higher risk scores showed slight deviations, particularly in BA.5 Omicron subset. The risk assessment model is not only adaptable for incorporating new evidence as SARS‐CoV‐2 subvariants emerge but also very valuable in facilitating the optimal individualized medical care for PCC patients and prioritizing a spectrum of risk groups for early PCC diagnosis. Notably, the adaptability of Bayesian DAG model enhances PCC risk prediction, enabling data integration for evolving SARS‐CoV‐2 contexts and informing healthcare resource allocation for high‐risk groups.

## 1 INTRODUCTION

It is of paramount importance to assess and predict an individual's risk of developing a post‐COVID‐19 condition (PCC). In spite of a number of studies addressing the possible risk factors associated with PCC that may be ex‐changeable with long‐COVID‐19 (Arjun et al., 2022; Sudre et al., 2021), evidence supporting such association studies has been individually reported by various SARS‐CoV‐2 variants in various countries and regions worldwide. Therefore, their external generalizability is rather limited and subject to the weakness of the small sample size. More importantly, each piece of information has not been synthesized in a systematic way. The recent meta‐analysis (Tsampasian et al., 2023) of integrating all kinds of association studies between risk factors and PCC not only revealed a panorama view of risk factors associated with PCC but also offered an opportunity for quantifying the potential of developing PCC on an individual level to streamline the stratification of a spectrum of risk groups in the underlying susceptible population with a constellation of factors in a systematic way. The tabular data provided from this study also creates a golden opportunity for building up a personalized risk assessment for PCC.

Meta‐analysis has been extensively used to synthesize data on a series of key interest parameters from multiple studies (Glass, 1976). The combined findings from the listed studies would be used to establish the summary of the effect size for each risk factor associated with the outcome of interest after pooling findings from various studies (Normand, 1999). However, a common challenge in many applications arises from the fact that different studies may include various sets of covariates that may or may not potentially overlap. Within a substantial consortium of epidemiological research, it is customary to assess certain crucial risk factors across all the investigations. However, it is inevitable that certain potentially significant covariates are only measured in a subset of the studies, rather than in all of them (Berkey et al., 1995; van Houwelingen et al., 2002). The meta‐regression analysis was adopted to accommodate a series of relevant covariates (Platt et al., 1999; St‐Pierre, 2001). Moreover, the biased estimation of parameters inherent from the heterogeneity across studies is supposed to be calibrated with the incorporation of the random effect across studies leveraging advanced meta‐analytics such as the generalized mixed model methodology. Although the mixed meta‐regression model extends beyond a basic synthesis of effect sizes and enables researchers to investigate the associations between study‐level variables (such as covariates) and the reported effect sizes in the included studies, it is still intractable to directly apply such a method to develop a series of composite risk scores (CRSs) synthesized from various studies, spanning over different periods with the common event‐based outcome but possibly with different underlying predictors varying with the time period, as seen in the current scenario of SASRS‐CoV‐2 variants and subvariants.

The PCC arising from a series of SARS‐CoV‐2 variants from wild type/D614G, Alpha, Delta, until Omicron in different time periods is a typical case. In addition to a systematic review and the conventional meta‐analysis method, a new statistical approach is therefore required to transcend various covariates from individual studies into a comprehensive risk assessment model in a synthetic manner to cover different periods of SARS‐CoV‐2 mutants. The development of Bayesian directed acyclic graphic (DAG) model is one of the useful approaches.

Based on the information provided from this comprehensive meta‐analysis of the Tsampasian et al. study, we sought to create a personalized risk score for assessing and forecasting the risk of developing PCC leveraging a Bayesian DAG model algorithm and validated the proposed risk score model with both real‐world data from Israeli and Japanese SARS‐CoV‐2.

## 2 METHODS

### 2.1 Data source

The risk score for PCC was calculated on the basis of the demographic characteristics and comorbidities in a meta‐analysis study (Tsampasian et al., 2023). It comprised 41 observational studies from the inception of COVID‐19 to December 5, 2022, including 860,783 adult patients with COVID19. An infected case with symptoms lasting longer than 3 months was considered PCC, consistent with the WHO definition in this study. The risk factors included female sex, advanced age (<40, 40–69, 70+), higher body mass index, smoking, preexisting conditions, prior hospitalization or ICU admission, and incomplete vaccination, giving 148 data records with 15 levels of risk factor.

### 2.2 Bayesian approach for a personalized risk score model for PCC

**FIGURE 1:**
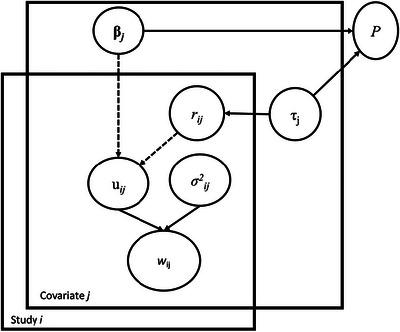
Bayesian directed acyclic graphic (DAG) model for estimating and predicting individual risk of post‐COVID‐19 condition (PCC).

To incorporate covariates relevant to the estimated effect size, the integration of different studies applying a generalized linear mixed model (e.g., generalized random‐intercept model) with a Bayesian approach was developed. The incorporation of the random intercept can account for unobserved variations across studies. Under this framework, we proposed a Bayesian DAG model to assess the clinical weight (effect size) of each risk factor on the risk of PCC while simultaneously accounting for fourteen risk factors (see Figure 1). The Bayesian DAG model applied to meta‐analysis has been demonstrated by Smith et al. (1995) and has been extensively used as one of generalized linear mixed models in literature (Lian et al., 2019; Yen et al., 2006). Here, we take advantage of the Bayesian approach with the incorporation of prior distributions for sharing information on all related covariates across studies with each other to cope with the missing covariates of each study and its flexibility of generating samples of posterior distribution to re‐construct sufficient individual data as if they were primarily collected. In brief, with the mean value and variance fluctuating with each risk factor across studies, the risk of PCC was transformed with the log (odds ratio) of each study to obey a normal distribution. The Bayesian DAG model underpinning the generalized linear random intercept model using the logit link as a link function was first constructed to derive the posterior distribution formed by the prior distribution in conjunction with the likelihood function given the conditional independence (d‐separation) circumstances of all the involved parameters. A normal distribution was adopted for the prior distribution of each regression coefficient. For the variance parameter, the prior distribution of the inverse gamma distribution was adopted. The parameters of all regression coefficients of relevant covariates were trained by applying the Bayesian Markov Chain Monte Carlo (MCMC) method to generate samples that were leveraged to approximate the estimates of the marginal posterior distribution following the ergodic property of the stationary distribution of the Markov chain over a series of iterations. The initial values for parameters were assigned with reference to the results of maximum likelihood estimates. To stabilize and eliminate the effects from the initial values, the first numbers of burn‐in iterations were discarded. A Gibbs sampling algorithm was employed to derive samples of a stationary posterior distribution by which inferences on parameters were made. The detailed algorithms for applying the Bayesian DAG model to our scenario of PCC are elaborated as follows:

1. The random variable $W_{ij}$, namely the dependent variable of PCC, associated with the jth risk factor of the ith study is expressed by $\log(\mathrm{OR}{\left(\mathrm{odds}\;\mathrm{ratio}\right)}_{ij})$, which obeys the normal distribution denoted as $\mathrm{Normal}(u_{ij},\sigma_{ij}^{2})$ where $u_{ij}$ and $\sigma_{ij}^{2}$ represent the corresponding mean value and variance. Each *W* was abstracted from each study enrolled in the meta‐analysis of PCC (Tsampasian et al., 2023).
2. The mean value of $u_{ij}$ as a function of risk factors was modeled by linking $u_{ij}$ with the estimated regression coefficients ($\beta_{j}$) of each fifteen categories of risk factors plus the random effect that captures the variance dependent on risk factor across studies. The equation is expressed as follows:
$$\;u_{ij}=\beta_{j}+r_{ij},$$

3. Assign noninformative prior distributions to each hyperparameter, including $\tau_{j}$ and $\beta_{j}$
$$\tau_{j}∼\mathrm{Gamma}\left(0.01,0.01\right)$$
$$\beta_{j}∼\mathrm{Normal}\left(0,10^{-6}\right)$$
4. The CRS is calculated using 2^15^combinations of risk factors.
$$\mathrm{CRS}=\left({\hat{Z}}_{1}X_{1}+{\hat{Z}}_{2}X_{2}+\cdots \cdots +{\hat{Z}}_{14}X_{14}+{\hat{Z}}_{15}X_{15}\right)\times 100$$

 where $r_{ij}$ follow a normal distribution denoted as $r_{ij}∼\mathrm{Normal}\left(0,\tau_{ij}\right)$

Ranging from 0 to 500 as shown in Table 1.

5. The predicted value denoted as $\hat{Z}$ for each risk factor was generated by the aforementioned normal distribution denoted as $\hat{Z}∼\mathrm{Normal}\left(\beta_{j},\tau_{j}\right)$
6. Plot the normal distribution of CRS.
7. The risk of developing PCC by three periods dominated by Wild/D614G, Alpha/Delta, and Omicron is predicted by a logistic regression equation expressed by the Equation (1):
(1) $$\mathrm{Probability}\;=\frac{1}{1+\exp\left(-\left(\alpha \mathrm{VOC}+\frac{\mathrm{CRS}}{100}\right)\right)}\;$$

**TABLE 1:** The decile of the composite risk score and the corresponding risk of post‐COVID‐19 condition (PCC) for quartile‐based risk groups.

Decile (%)	Composite risk score	Risk of PCC	Quartile‐based risk groups
90–100	350–500	≥90%	Very high risk group (75th–100th)
80–89	310–349	73%–89%	
70–79	284–309	54%–72%	
			High risk group (50th–75th)
60–69	263–283	37%–53%	
50–59	241–262	23%–36%	
40–49	220–240	13%–22%	Moderate risk group (25th–50th)
30–39	198–219	7%–12%	
20–29	173–197	3%–6%	
			Low risk group (<25th)
10–19	140–172	1%–2%	
<10	<140	<1%	

The intercept, α, was estimated as −9.056 for wild/D614G/Alpha, −9.532 for Delta, and −10.717 for Omicron. All these intercepts refer to the previous studies (Antonelli et al., 2022; Willan et al., 2023) across three corresponding periods of SARS‐CoV‐2 variants.

8. After discarding 10,000 burn‐in samples, another 10,000 samples with Gibbs sampling methodology were used to estimate the parameters of interest based on their posterior distributions. The MCMC simulation was carried out using WinBugs (Cambridge MRC). The risks of PCC for three eras of SARS‐CoV‐2 variants were plotted accompanied by the normal distribution of risk score. The predictive distribution (denoted by P in Figure 1) based on *β_j_* and *τ_j_* was also generated by using MCMC simulation for obtaining Bayesian predictive interval.

The languages of the Bayesian DAG algorithm are depicted as follows:

While *m* ≤ 183, do /*m represents available data record*/

Read data of each mean value and variance of the *j*th risk factor in the *i*th study for $W_{ij}$ following $\mathrm{Normal}(u_{ij},\sigma_{ij}^{2})$

If *m* > 183 then break loop

While *i*≤ 47, do

While *j* ≤ 15, do

$$\;u_{ij}=\beta_{j}+r_{ij}$$

$r_{ij}$ follows $\mathrm{Normal}(0,\tau_{ij})$

 if *j* > 15 then break loop

 if *i* > 47 then break loop

While *j* ≤ 15, do

$\beta_{j}$ follows Normal(0,0.000001)

${\hat{Z}}_{j}$ follows $\mathrm{Normal}(\beta_{j},\tau_{j})$ /* Ẑ*_j_* represents the predicted weight*/

$OR_{j}$ = exp($\beta_{j}$)

Predictive $OR_{j}$= exp(${\hat{Z}}_{j}$)

$\tau_{j}$ follows Gamma (0.01, 0.01)

If *j* > 15 then break loop

A CRS was created by adding up all the estimated clinical weights given the 2^15^ combinations of each category of risk factor. The underlying susceptible population was stratified using the decile risk score. The risk of PCC given each risk score was therefore computed and predicted making allowance for the baseline risk varying with three eras of SARS‐CoV‐2 variants (Wild/D614G/Alpha, Delta, and Omicron BA.1/BA.2).

### 2.3 Model validation

To illustrate our proposed personalized risk score and extend its applications to assess the risk of developing PCC in different datasets, we utilized data obtained from two studies (Iba et al., 2024; Kuodi et al., 2022) for external validation. The study of Kuodi et al. included Israeli adults aged 18 and over who had been infected by COVID‐19 in the period between March 2020 and November 2021 before the Omicron subvariant. Information regarding age, sex, vaccine status, hospitalization, preexisting health conditions, and post‐COVID‐19 symptoms was collected through self‐reported questionnaires. Individuals who experienced any of the reported symptoms were categorized as having PCCs in our analysis. Individual data used for model validation are presented in Table A1. Another study used for external validation is pertaining to Japanese adults aged between 18 and 69 who had been infected by COVID‐19 during the BA.5 Omicron‐dominated period. Self‐reported information regarding age, sex, vaccine status, body mass index, preexisting health conditions, and PCC was collected through the questionnaire survey. Individual data generated from the study of Iba et al. for model validation are presented in Table A2. The goodness of fit for model validation between the observed probability and the predicted probability pursuant to posterior‐predictive distribution was performed with a Bayesian chi‐square test (Johnson, 2004).

The Bayesian $X^{2}$ statistic $R(\hat{\theta })$ following the notation of Johnson is expressed by Equation (2):

(2) $$R\left(\hat{\theta }\right)=\sum_{i=1}^{I}{\left[\frac{O_{i}\left(\hat{\theta }\right)-np_{i}\left(\hat{\theta }\right)}{\sqrt{np_{i}\left(\hat{\theta }\right)}}\right]}^{2},\mathrm{df}=I-1$$

$O_{i}$ represents the number of observations that fell into the ith bin. The number of bins, say *I*, are determined by the quantiles of risk score distribution, denoted as $\hat{\theta }$. $p_{i}$ represents the predictive probability assigned in the interval *i* over the sample space *n*. Under the regular condition, the distribution of $R(\hat{\theta })$ is assumed to converge to $X^{2}$ with *I −* 1 degree of freedom as *n* → ∞ to have an asymptotic distribution of $R(\hat{\theta })$.

## 3 RESULTS

### 3.1 The personalized risk score for PCC

The self‐assessed personalized risk score for developing PCC based on Equation (1) is constructed as follows:

CRS =

$$\begin{array}{ccc} & & 44\times \mathrm{if}\;\left(\mathrm{female}\right)+23\times \mathrm{if}\left(40\leq \mathrm{age}\leq 69\right)+17\times \mathrm{if}\left(\mathrm{age}\geq 70\right)\\ & & +19\times \mathrm{if}\left(\mathrm{BMI}>30\right)+15\times \mathrm{if}\left(\mathrm{current}\;\mathrm{smoker}\right)\;+88\\ & & \times \mathrm{if}\left(\mathrm{previously}\;\mathrm{hospitaliazation}\;\mathrm{for}\;\mathrm{COVID}-19\right)\;+73\\ & & \times (\mathrm{ICU}\;\mathrm{administration}\;\mathrm{for}\;\mathrm{COVID}-19)\;+57\\ & & \times \mathrm{if}\left(\mathrm{incomplete}\;\mathrm{vaccination}\right)+17\times \mathrm{if}\left(\mathrm{anxiety}\;\mathrm{or}\;\mathrm{depression}\right)\\ & & +21\times \mathrm{if}\;\left(\mathrm{asthma}\right)+10\times \mathrm{if}\;\left(\mathrm{CKD}\right)+31\times \mathrm{if}\;\left(\mathrm{COPD}\right)\\ & & +4\times \mathrm{if}\left(\mathrm{diabetes}\right)+50\times \mathrm{if}\;\left(\mathrm{immunosuppression}\right)\\ & & +27\times \mathrm{if}\;\left(\mathrm{ischemic}\;\mathrm{heart}\;\mathrm{disease}\right) \end{array}$$

**FIGURE 2:**
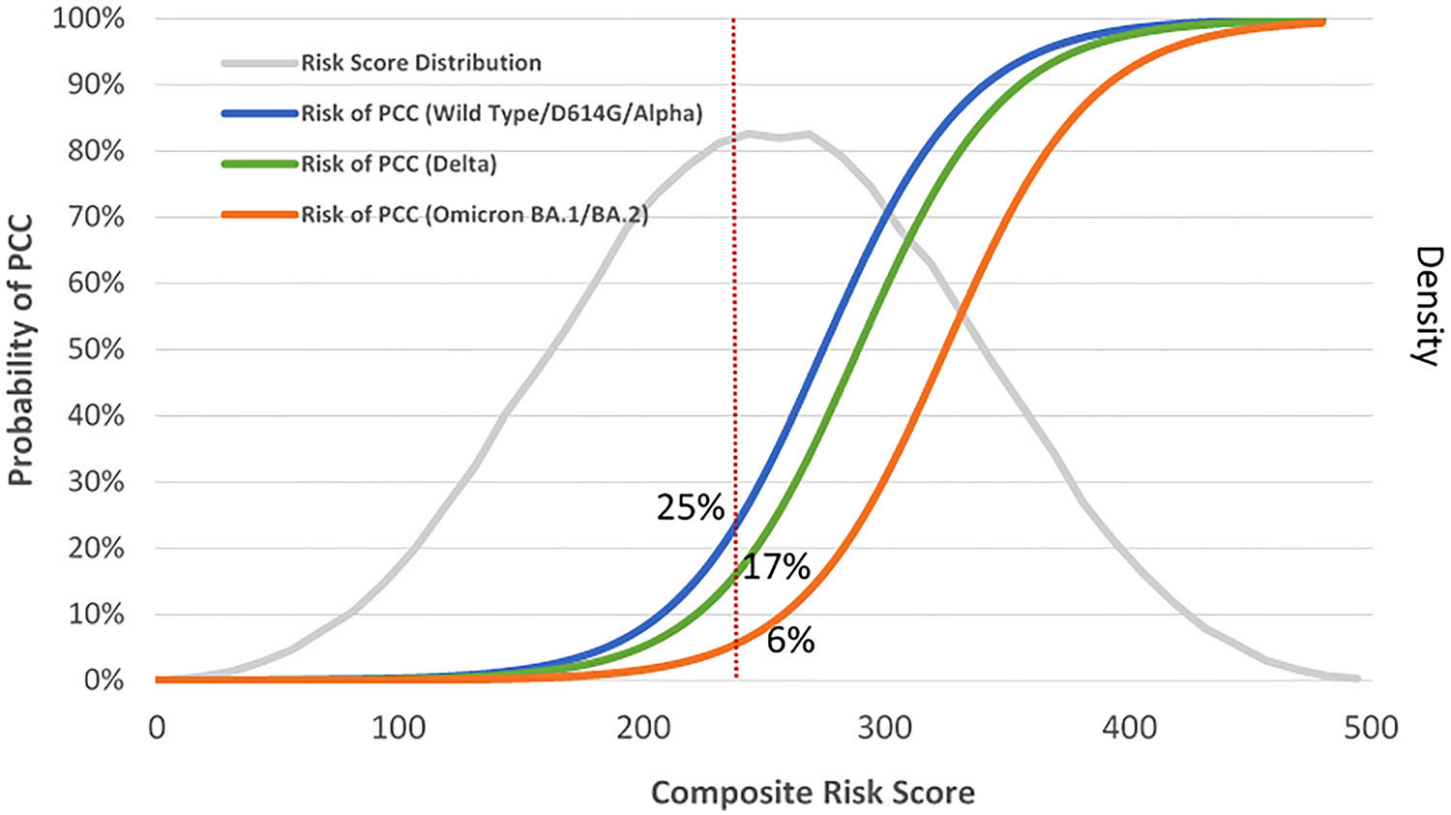
Composite risk score distribution and the posterior probability of post‐COVID‐19 condition (PCC).

Note that the clinical weights refer to the estimates of Bayesian posterior odds ratios. The score ranged from 0 to 500 with the corresponding normal distribution as shown in Figure 2 (lower panel). We divided the patient population into four quartile‐based risk groups.

Table 1 shows the CRS and the risk of PCC in deciles from <1% for the lowest decile to ≥90% for the 90th highest decile. The score ranged from 0 to 500, yielding the normal distribution as shown in Figure 2 (lower panel). The 90th highest risk patients were 90 times the risk for PCC compared with the 10th lowest‐risk patients.

The risk of having PCC was around 23% for the average‐risk group (50th) during the entire period. The corresponding figures were 25% for Wild/D614G/Alpha, 17% for Delta, and 6% for Omicron.

### 3.2 External validation of PCC

#### 3.2.1 The Israel study before omicron

**FIGURE 3:**
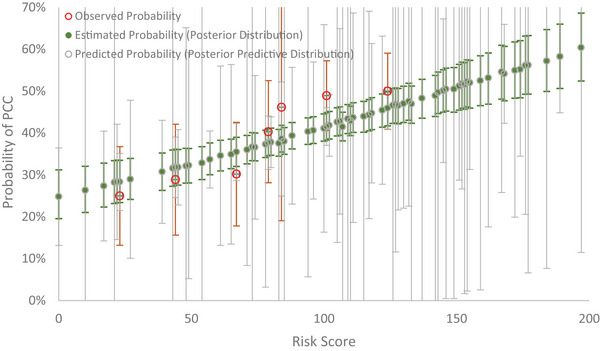
Observed, estimated, and predictive probability of post‐COVID‐19 condition (PCC) by risk score using Israel data.

The prevalence of PCC in Israel before Omicron was 40% (397/951). In this external data, infected cases with incomplete vaccines, asthma, chronic kidney disease, chronic obstructive pulmonary disease, diabetes, or immunosuppression were more likely to develop PCC. Among the total of 15 predictors, 9 risk factors in this dataset could be applied to the predictive model. After applying the clinical weights into individual variables, the average risk score was 90 in this dataset. The observed probability, the estimated probability with the posterior distribution (green color), and the predictive probability with the posterior‐predictive distribution (gray color) by risk score are delineated in Figure 3. Note that the 95% credible intervals of the predicted probability were wider than the estimated probability because the former has integrated out all relevant parameters according to posterior‐predictive distribution following the Johnson (2004) study. The goodness‐of‐fit test shows a lack of statistical discrepancy between the observed and the predictive probabilities following Equation (2) ($X^{2}=3.27$, df = 6, *p* = 0.7734).

#### 3.2.2 Japanese Omicron BA.5 study

**FIGURE 4:**
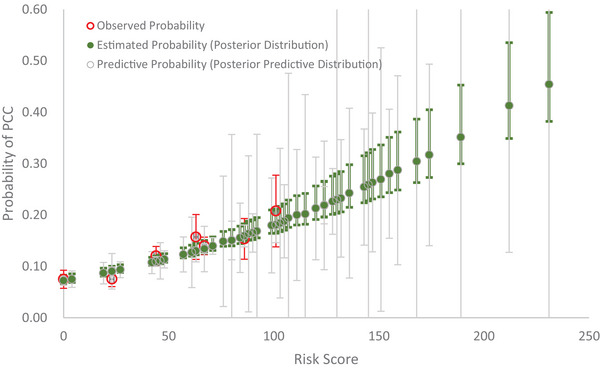
Observed, estimated, and predictive probability of post‐COVID‐19 condition (PCC) by risk score using Japan data.

The prevalence of PCC in Japanese external data for Omicron BA.5 was 11.8% (992/8392). Six risk factors including age, sex, body mass index, vaccine status, severity of COVID‐19, and underlying medical conditions in this dataset were applied to the predictive model. Figure 4 shows the observed probability, the estimated probability with the posterior distribution (green color), and the predictive probability with the posterior‐predictive distribution (gray color) by risk score. The result of the goodness‐of‐fit test shows a lack of statistical discrepancy between the observed and the predictive probabilities following Equation (2) ($X^{2}=7.16$, *p* = 0.3059).

**FIGURE 5:**
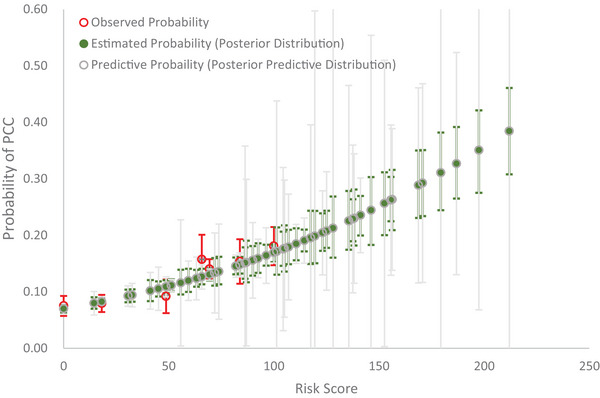
Observed, estimated, and predictive probability of post‐COVID‐19 condition (PCC) by risk score with prior information using Japan data.

The model fitting was further improved if we incorporated the covariates with informative prior coefficients (sex∼Normal(0.44,0.0026), age∼Normal(0.23,0.0113), BMI∼Normal(0.19,0.0028), severeCOVID19∼Normal(0.88,0.0253), novaccine∼Normal(0.57,0.0315), and underlyingmedicalconditions∼Normal(0.4,0.0043)) into the predictive model. With all these informative priors, Figure 5 shows an even better model prediction with a smaller Bayesian statistic of goodness‐of‐fit ($X^{2}=5.16$, *p* = 0.5236).

## 4 DISCUSSION

Before the advent of the meta‐analysis, it was very difficult to assess and predict an individual risk for developing PCC partly because each risk factor had been individually reported with small sample size and partly because the results of previous studies are heterogeneous as they have spanned across various SARS‐CoV‐2 variants and regions worldwide. Taking advantage of information derived from a comprehensive meta‐analysis, we proposed a personalized risk assessment model for the risk of PCC using the Bayesian DAG method.

The merits of applying the Bayesian DAG approach to data from the recent well‐conducted meta‐analysis are threefold. First, it is easy to derive a self‐assessed personalized risk score for PCC for each individual given his/her own risk factors. Second, the posterior distribution can be used to estimate an individual's risk for PCC given the currently observed data whereas the predictive distribution can forecast an individual's risk for PCC while the new data provided. Both tackle the uncertainty of an individual's risk for PCC with the 95% posterior and predictive credible intervals. Third, the proposed Bayesian approach is very flexible in accommodating the incorporation of new evidence on PCC in the era of new SARS‐CoV‐2 subvariants such as XBB.1.15 or JN.1 as externally validated in Japanese Omicron BA.5. Finally, the proposed personalized risk score is useful for facilitating optimal individualized medical care for patients with PCC as well as prioritizing the risk groups for early PCC diagnosis using COVID‐19 related biomarker screening (Galán et al., 2022; Lai et al., 2023).

The proposed personalized risk‐score model was also applied to the external datasets from Israel and Japan. The predicted probability of PCC using both Israel and Japan data was well comparable with the one derived from our risk assessment model. As our predictive model was developed based on data before BA.2 Omicron, the risk factors for PCC might have some differences for BA.5 Omicron. It could be argued that the calibration between the observed and predicted probabilities in external data from Japan may be limited to the availability of predictors and the unexplained variation due to the emergence of new variant concerns, such as Omicron BA.5. To improve this weakness, we incorporated the informative prior coefficients to demonstrate the improvement in the calibration between observed and predicted probabilities ($X^{2}$ reduced from 7.16 to 5.16). Thus, we believe that while the healthcare of long COVID has been neglected, particularly in low‐ and middle‐income countries (Ledford, 2024), our Bayesian risk assessment model may aid health decision‐makers in identifying the high‐risk group and allocating the medical resources for long COVID care.

Using Bayesian DAG random‐effect model has several advantages. Random‐effect analysis accounts for the heterogeneity across studies derived from measured and unobserved factors that are consistent within each study but vary across studies. Leveraging these random effect reduced the random error and enhanced the accuracy of the model. Second, it can also improve the robustness of estimates by combining information across studies, even with limited data in some studies. To elucidate these effects, we examined the effects of risk factors on PCC using the same data with the random‐effect model compared with the fixed‐effect model. The resultant factor coefficients are shown in Table B1. The differences in deviance information criterion (DIC) test across a series of random‐effect modes and the corresponding estimated random effects ($\tau_{j}$) incorporating with one‐by‐one covariates are presented in Table B2. The results regarding the effect size of random effects seems robust across forward model selection process. This may suggest that the heterogeneity across studies was consistent once the random effect was considered and relevant significant covariates were captured in the model. However, the fixed‐effect model yielded a DIC (deviance information criterion) of 1173.3, which was higher than the DIC of −14.350 produced by the random‐effect model. These findings have justified the necessity of incorporating the random effect for making allowance for the heterogeneity across studies. Our random‐effect model is robust to the forward model selection varying with different covariates.

As DIC values, when comparing multiple models, provide a relative measure rather than the absolute value a smaller DIC indicates the better model fit. Since the number of studies contributing to each factor varies, we utilized the forward random‐effect model selection, starting from 7 factors and increasing up to 15 factors. The DIC values changed from −10.309 to −30.252 throughout this process. The forward one‐by‐one addition of covariates highlights how each factor contributes to explaining the variability in the outcome (Table B3). Most covariates, except for ischemic heart disease, significantly improved the model. Although the DIC shows slight differences in random‐effect models with different factor loadings, the estimated coefficients for each covariate were still robust.

**FIGURE C1:**
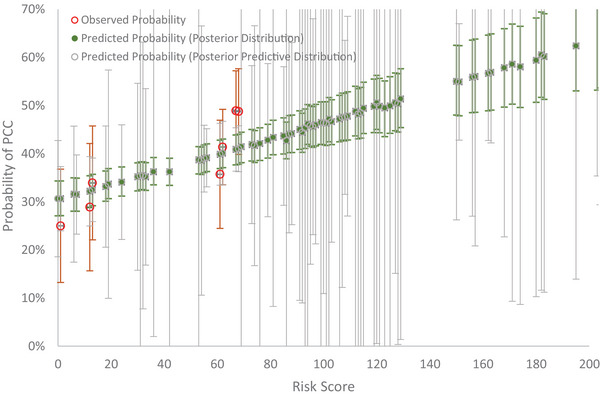
Observed, estimated, and predictive probability of post‐COVID‐19 condition (PCC) with risk scores by fixed‐effect model in Israel data.

**FIGURE C2:**
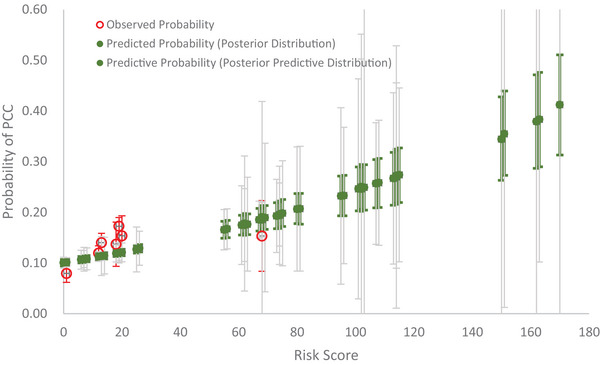
Observed, estimated, and predictive probability of post‐COVID‐19 condition (PCC) with risk scores by fixed‐effect model in Japan data.

In the fixed‐effect model, the observed probabilities in the Israel data appear more clustered into two groups (lower and higher risk scores) (Figure C1). The goodness‐of‐fit test revealed a moderate fit compared to the alignment between observed and predicted probabilities in the random‐effect model. An even poorer model fit ($X^{2}=38.19$, df = 6, *p* < 0.0001) was observed when the predictive model was built based on the estimates from the fixed‐effect model (Figure C2).

However, the external validation of the Bayesian DAG random‐effect model revealed slight discrepancies between observed and predicted probabilities, particularly at higher risk scores, in both Israeli and Japanese datasets. Beyond the potential influence of evolving viral strains, real‐world data for high‐risk groups typically involve smaller sample sizes, resulting in greater variation and increasing susceptibility to bias, making accurate prediction challenging due to insufficient data.

We attempted to address this issue by applying Bayesian priors derived from meta‐analysis to Japanese data; however, the use of priors still carries one limitation that the unobserved heterogeneity beyond the explanation from covariates inherent from the random effect in the meta‐analysis has been not introduced to priors. For example, we acknowledge that assumptions embedded in prior distributions may not fully capture the rapidly evolving dynamics of COVID‐19 variants and the availability of diverse datasets. This section examines how prior selection influences model performance and suggests that incorporating recent data with adaptable priors could improve predictive accuracy.

While the random‐effect models offer better accuracy than the fixed‐effect models as discussed above, applying the random‐effect model to predicting the risk of PCC for the broader population is of great interest by providing sensitivity analysis based on Tables B1 and B2 for the comparisons between the fixed‐effect models and the random effects. However, although the random‐effect model can throw light on the heterogeneity across studies as demonstrated in Table B2 to give a cue to providing the rationales for uncovering those unexplained heterogeneity, they are still limited to offer new insights into how other specific factors such as the rapid evolution of SARS‐CoV‐2 variants, the measurements of PCC, the repeated infection in the same individual, and the underlying features of healthcare delivery system improve the better accuracy for prediction. Accordingly, the potential limitations of the current model based on meta‐analysis data while applying to external populations should be improved by uncovering new information as related to the heterogeneity across studies as shown in these random‐effect models. Continuous model validation and recalibration using updated datasets like our extension from Israel to Japan are necessary to ensure robustness and generalizability across diverse settings in the world.

In conclusion, given the 148 data records with 15 levels of risk factor from meta‐analysis, we developed a personalized risk assessment model by Bayesian DAG approach for estimating and forecasting the risk of developing PCC. The feasibility of its application to risk stratification would be useful for facilitating optimal individualized medical care for PCC patients.

## Data Availability

The data that support the findings of this study are available from the corresponding author upon reasonable request.

## APPENDIX A

### A.1

**TABLE A1:** Post‐COVID conditions (PCC) among infected cases during the Wild/D614G and Alpha variant of concern (VOC)‐dominant period in Israel.

Risk factors	Non‐PCC		PCC	
	*N* = 572	%	*N* = 379	%
Age				
<40	223	39.0	160	42.2
40–69	291	50.9	169	51.7
≥70	58	10.1	23	6.1
Gender				
Male	192	42.8	91	30.2
Female	257	57.2	210	69.8
Hospitalization				
No	517	90.4	348	91.8
Yes	55	9.6	31	8.2
Vaccine				
No	212	37.1	82	21.6
Complete	360	62.9	297	78.4
Asthma				
No	558	97.5	352	94.2
Yes	14	2.5	22	5.8
CKD				
No	571	99.8	371	97.9
Yes	1	0.2	8	2.1
COPD				
No	566	98.9	370	97.6
Yes	6	1.1	9	2.4
Diabetes				
No	546	95.5	344	90.8
Yes	26	4.5	35	9.2
Immunosuppression				
No	559	97.7	358	94.5
Yes	13	2.3	21	5.5
Risk score (mean ± SD)	84.4 ± 44.9		99.0 ± 41.7	
Minimum risk score	0		0	
Maximum risk score	239		262	

Abbreviations: CKD, chronic kidney disease; COPD, chronic obstructive pulmonary disease; SD, standard deviation.

**TABLE A2:** Post‐COVID conditions (PCC) among infected cases during the Omicron BA.5‐dominant period in Japan.

Risk factors	Non‐PCC		PCC	
	*N* = 7400	%	*N* = 992	%
Age				
<40	3253	44.0	400	40.3
40–69	4147	56.0	592	59.7
Gender				
Male	3272	44.2	288	29.0
Female	4128	55.8	704	71.0
BMI				
<18.5	766	10.4	132	13.3
18.5–25	5408	73.1	693	69.9
>25	1226	16.6	167	16.8
Underline medical conditions				
No	5716	77.2	729	73.5
Yes	1684	22.8	263	26.5
Severity of COVID‐19				
Asymptomatic/mild	7336	99.1	972	98.0
Moderate/severe	64	0.9	20	2.0
Vaccine				
No	584	7.9	107	10.8
Yes	6816	92.1	895	89.2
With noninformative prior				
Risk score (mean ± SD)	46.8 ± 31.0		57.1 ± 31.9	
Minimum risk score	0		0	
Maximum risk score	212		231	
With informative prior				
Risk score (mean ± SD)	52.4 ± 33.9		64.5 ± 33.9	
Minimum risk score	0		0	
Maximum risk score	205		218	

Abbreviations: BMI, body mass index; SD, standard deviation.

## APPENDIX B

### B.1

**TABLE B1:** Estimated coefficients by fixed‐effect and random‐effect models incorporating with different covariates.

		Random effects									Fixed effects
Variables	No. of studies	7 Covariates	8 Covariates	9 Covariates	10 Covariates	11 Covariates	12 Covariates	13 Covariates	14 Covariates	15 Covariates	15 Covariates
		Coef. (SD)	Coef. (SD)	Coef. (SD)	Coef. (SD)	Coef. (SD)	Coef. (SD)	Coef. (SD)	Coef. (SD)	Coef. (SD)	Coef. (SD)
Gender (female vs. male)	38	0.449 (0.05)	0.440 (0.05)	0.438 (0.05)	0.444 (0.05)	0.437 (0.05)	0.442 (0.05)	0.447 (0.05)	0.444 (0.05)	0.441 (0.05)	0.107 (0.01)
40 ≤ age ≤ 69 vs. age <40	12	0.239 (0.11)	0.243 (0.11)	0.218 (0.15)	0.197 (0.13)	0.262 (0.12)	0.20 (0.15)	0.257 (0.12)	0.253 (0.12)	0.234 (0.11)	0.005 (0.01)
Age ≥ 70 vs. age < 40	13	0.187 (0.14)	0.139 (0.13)	0.209 (0.14)	0.151 (0.17)	0.148 (0.15)	0.159 (0.15)	0.168 (0.15)	0.173 (0.15)	0.168 (0.12)	0.183 (0.014)
Current smoker	21	0.158 (0.06)	0.153 (0.07)	0.153 (0.06)	0.170 (0.07)	0.157 (0.06)	0.153 (0.06)	0.151 (0.07)	0.162 (0.06)	0.159 (0.06)	0.094 (0.01)
Vaccination	4							−0.569 (0.19)	−0.568 (0.21)	−0.567 (0.18)	−0.548 (0.04)
BMI > 30	16	0.170 (0.065)	0.157 (0.06)	0.154 (0.05)	0.157 (0.05)	0.17 (0.06)	0.176 (0.07)	0.177 (0.08)	0.173 (0.05)	0.165 (0.05)	0.074 (0.01)
Asthma	13	0.207 (0.07)	0.208 (0.07)	0.202 (0.07)	0.205 (0.08)	0.208 (0.07)	0.201 (0.07)	0.213 (0.06)	0.198 (0.08)	0.214 (0.08)	0.196 (0.01)
COPD	9			0.28 (0.12)	0.287 (0.15)	0.288 (0.15)	0.270 (0.14)	0.289 (0.13)	0.302 (0.14)	0.312 (0.14)	0.334 (0.02)
Diabetes	18	0.052 (0.07)	0.05 (0.07)	0.046 (0.07)	0.030 (0.07)	0.038 (0.07)	0.041 (0.07)	0.040 (0.07)	0.047 (0.07)	0.039 (0.07)	0.059 (0.02)
CKD	8					0.095 (0.11)	0.113 (0.12)	0.108 (0.13)	0.114 (0.10)	0.098 (0.11)	0.190 (0.02)
Ischemic heart disease	5						0.269 (0.15)	0.26 (0.16)	0.267 (0.16)	0.277 (0.18)	0.249 (0.04)
Immunosuppression	3									0.505 (0.41)	0.405 (0.18)
Anxiety	4								0.188 (0.11)	0.174 (0.10)	0.218 (0.01)
Previously hospitalization	8				0.906 (0.14)	0.887 (0.17)	0.902 (0.16)	0.907 (0.16)	0.899 (0.17)	0.88 (0.16)	0.949 (0.02)
ICU administration for COVID‐19	10		0.730 (0.12)	0.737 (0.12)	0.737 (0.13)	0.736 (0.12)	0.743 (0.12)	0.734 (0.12)	0.74 (0.12)	0.731 (0.12)	0.862 (0.04)

Abbreviations: BMI, body mass index; CKD, chronic kidney disease; COPD, chronic obstructive pulmonary disease; ICU, intensive care unit.

**TABLE B2:** Estimated random effects, $\tau_{j}$, across studies and chi‐square (DIC) in random‐effect analysis incorporating with different covariates.

Variables	No. of studies	Adding one‐by‐one covariate forward random‐effect model								
		7	8	9	10	11	12	13	14	15
		Effect size of random effect								
		Mean (SD)	Mean (SD)	Mean (SD)	Mean (SD)	Mean (SD)	Mean (SD)	Mean (SD)	Mean (SD)	Mean (SD)
Gender (female vs. male)	38	21.15 (6.8)	21.13 (6.9)	21.21 (6.9)	20.98 (6.7)	21.31 (6.8)	21.18 (6.9)	21.24 (6.8)	21.33 (6.9)	21.41 (6.9)
40 ≤ age ≤ 69 vs. age < 40	12	14.49 (9.3)	14.36 (9.1)	13.36 (9.0)	13.84 (8.9)	14.32 (9.3)	13.27 (8.7)	14.25 (9.1)	14.18 (9.0)	14.68 (9.4)
Age ≥ 70 vs. age < 40	13	20.53 (18.5)	20.9 (18.0)	20.32 (17.7)	19.12 (17.9)	21.02 (19.2)	20.59 (18.4)	20.82 (018.8)	20.1 (18.9)	21.76 (19.1)
Current smoker	21	110.1 (89.2)	110.1 (88.9)	110.3 (86.9)	102.3 (87.3)	108.2 (87.0)	112.6 (91.8)	112.0 (90.9)	108.0 (88.2)	113.0 (91.1)
Vaccination	4							17.15 (15.9)	16.18 (15.5)	17.27 (15.9)
BMI > 30	16	54.56 (35.0)	55.64 (35.5)	57.87 (36.1)	64.51 (37.4)	55.21 (36.3)	55.34 (36.8)	53.47 (35.8)	54.41 (35.5)	55.3 (35.53)
Asthma	13	91.02 (73.2)	85.95 (68.1)	85.27 (69.2)	81.71 (66.7)	86.4 (69.5)	85.17 (68.0)	88.21 (71.2)	85.02 (68.4)	85.45 (68.8)
COPD	9			22.92 (20.0)	21.88 (20.7)	21.37 (19.1)	21.32 (18.2)	22.35 (19.7)	21.95 (19.5)	20.5 (18.3)
Diabetes	18	84.55 (67.1)	83.23 (67.5)	86.55 (70.1)	86.19 (66.4)	86.87 (71.1)	86.29 (68.3)	86.62 (68.1)	85.3 (67.4)	90.16 (70.6)
CKD	8					52.9 (52.5)	52.59 (54.0)	51.8 (53.8)	57.26 (56.3)	53.52 (56.2)
Ischemic heart disease	5						59.25 (65.6)	57.53 (65.1)	56.95 (63.2)	59.07 (68.9)
Immunosuppression	3									28.71 (46.7)
Anxiety	4								42.91 (40.7)	43.33 (40.1)
Previously hospitalization	8				10.35 (7.8)	9.84 (7.9)	10.1 (7.7)	9.9 (7.6)	9.65 (7.4)	10.03 (7.8)
ICU administration for COVID‐19	10		49.31 (52.7)	49.81 (50.5)	50.13 (52.8)	50.71 (53.6)	53.8 (53.3)	50.01 (52.5)	50.99 (53.7)	48.52 (50.8)

Abbreviations: BMI, body mass index; CKD, chronic kidney disease; COPD, chronic obstructive pulmonary disease; ICU, intensive care unit.

**TABLE B3:** A stepwise comparison of models by adding covariate.

Model	Adding covariate	DIC	*p*‐value
7 covariates		−22.253	
8 covariates	7 covariates + ICU administration for COVID‐19	−15.770	0.011
9 covariates	8 covariates + COPD	−11.998	0.052
10 covariates	9 covariates + Previously hospitalization	−30.252	<0.0001
11 covariates	10 covariates + CKD	−10.336	<0.0001
12 covariates	11 covariates + ischemic heart disease	−10.309	0.8695
13 covariates	12 covariates +vaccination	−14.364	0.044
14 covariates	13 covariates + anxiety	−24.490	0.0015
15 covariate	14 covariates + immunosuppression	−14.350	0.0015

Abbreviations: CKD, chronic kidney disease; COPD, chronic obstructive pulmonary disease; ICU, intensive care unit.
